# Chemoselective depolymerization of polyurethane *via* solvent-engineered glycolysis

**DOI:** 10.1039/d6ra05937f

**Published:** 2026-08-07

**Authors:** Sajid Hussain

**Affiliations:** a Department of Industrial Engineering, University of Padova Via Marzolo 9 35131 Padova Italy sajid.hussain@unipd.it

## Abstract

The chemical upcycling of polyurethane (PU) into virgin-quality feedstocks is hindered by toxic toluene diamine (TDA) formation during high-temperature glycolysis. Decoupling selective urethane cleavage from secondary thermolytic pathways remains a central engineering challenge. Here, we present a chemoselective depolymerization strategy utilizing solvent-system synergy. Systematic evaluation of binary-glycol environments identifies a 6 : 4 monoethylene-to-diethylene glycol ratio that balances high nucleophilicity with moderated oligomer solvation. By mapping the multivariable operational space, we define a constrained kinetic window—comprising 1 : 1 glycol-to-PU ratio, 1 mM diethanolamine catalyst, and a 160 °C thermal threshold—that maximizes urethane scission while arresting hard-segment fragmentation. This framework produces high-quality, single-phase glycolysate with minimized TDA concentrations (2278 mg kg^−1^). Predictive toxicological modeling confirms this refined product enables up to 40% substitution into secondary PU formulations without exceeding acute aquatic mortality thresholds. Integrating molecular-level solvent design with macroscopic process control provides a scalable, low-toxicity pathway for commercially viable circularity of polyurethane materials.

## Abstract 2

Solvent-engineered glycolysis (MEG : DEG = 6 : 4, 160 °C) mitigates toxic TDA generation during polyurethane upcycling, lowering TDA to 2278 mg kg^−1^ and enabling 40% polyol reuse in new foam.

## Graphical abstract

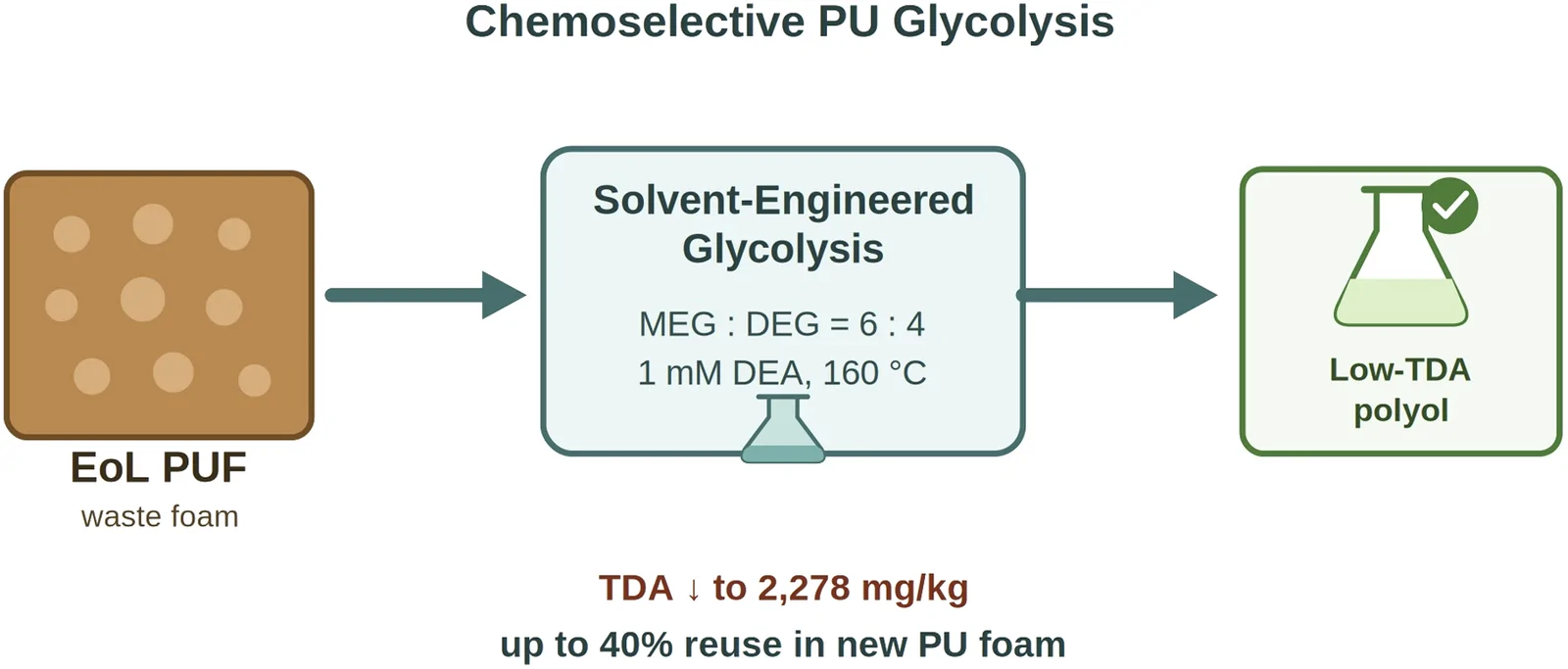

## 1 Introduction

The global production of polyurethane (PU) has surpassed 25 million metric tons annually, solidifying its status as one of the most indispensable polymer classes in modern industry.^1^ Its unique ability to be engineered into flexible foams, rigid thermal insulators, high-performance elastomers, and durable coatings has made it a staple in the automotive, furniture, and construction sectors.^2,3^ However, the very structural properties that make PU desirable—specifically the highly stable carbamate (urethane) and urea linkages in its cross-linked thermoset matrix—render it exceptionally resistant to natural degradation.^4^ As a result, the management of end-of-life (EoL) polyurethane foam (PUF) has become a primary environmental bottleneck. Traditional disposal methods, such as landfilling, are increasingly restricted due to land scarcity and the long-term risk of soil contamination from leaching additives.^5^ Incineration, while offering energy recovery, is plagued by the release of hazardous nitrogen-containing effluents, including hydrogen cyanide and nitrogen oxides, which necessitate complex and costly scrubbers to meet tightening air-quality regulations.^6,7^

In response to the limitations of mechanical recycling, which typically results in “downcycling” to low-value fillers, chemical recycling has emerged as the definitive pathway for establishing a true circular economy for PUFs.^8,9^ By depolymerizing the polymer chains back into their constituent precursors, chemical routes offer the potential to regenerate “virgin-quality” polyols.^10,11^ Among the various chemical modalities—including hydrolysis, acidolysis, aminolysis, and alcoholysis—glycolysis has garnered the most significant industrial interest.^12,13^ Glycolysis utilizes low-molecular-weight glycols to facilitate transesterification of the urethane bonds, producing a liquid glycolysate that can theoretically substitute polyols in new foam formulations.^14^ Recent advances in chemical recycling have demonstrated that rational solvent engineering has emerged as an effective strategy for directing depolymerization pathways, enhancing bond selectivity, and improving the recovery of high-value products from polymeric waste.^15,16^ In particular, recent studies have shown that carefully designed solvent environments can regulate reaction kinetics, stabilize reactive intermediates, and suppress undesired side reactions during solvent-promoted depolymerization processes.^17^ These findings underscore that the solvent is not merely a reaction medium but an active component governing depolymerization efficiency and product selectivity.^18^ The present work applies a binary glycol solvent-engineering strategy to polyurethane glycolysis, where the complementary physicochemical properties of monoethylene glycol and diethylene glycol are exploited to balance nucleophilic urethane bond cleavage with oligomer solvation, thereby minimizing secondary thermolytic degradation and suppressing the formation of toxic aromatic amines. Despite its conceptual simplicity, the industrial scale-up of PUF glycolysis is hindered by a persistent technical challenge: the formation of primary aromatic amines (PAAs), most notably toluene diamine (TDA).

The generation of TDA during glycolysis is not a primary reaction but a consequence of secondary thermolytic degradation. At the elevated temperatures required for liquefaction, the carbamate bond can undergo competitive cleavage pathways.^19^ While the desired nucleophilic attack by the glycol leads to polyol recovery, thermal dissociation and the presence of trace water can promote the formation of amines.^20–22^ TDA is a suspected human carcinogen and a potent aquatic toxin, and its presence in the recycled polyol poses severe risks.^23^ From an engineering perspective, TDA impairs the reactivity and cross-linking density of secondary PU products, leading to inferior mechanical properties.^24,25^ Furthermore, the removal of TDA *via* vacuum distillation or chemical quenching is energy-intensive and often reduces the total yield, thereby undermining the economic and environmental viability of the recycling process.^26,27^

To date, most glycolysis studies have relied on single-solvent systems, such as pure monoethylene glycol (MEG) or diethylene glycol (DEG). However, single-glycol systems present a “selectivity–severity” paradox.^28^ High-nucleophilicity glycols like MEG often require excessively high pressures or temperatures to maintain solubility, while high-boiling solvents like DEG may lack the nucleophilic “punch” needed for rapid urethane scission, leading to longer residence times that favor amine formation.^29,30^ Decoupling these kinetic pathways requires a sophisticated approach to solvent engineering that can independently tune the nucleophilic attack and the solvation environment.^31,32^ Additionally, the choice of catalyst is paramount. While organometallic catalysts like tin octoate or titanium alkoxides are effective, they often exhibit low selectivity and can remain as contaminants in the final product, affecting the properties of the reconstituted foam.^33,34^

In this work, we propose a novel engineering framework that utilizes a synergistic binary-glycol system catalyzed by diethanolamine (DEA) to achieve chemoselective depolymerization. By blending MEG and DEG in a specific 6 : 4 ratio, we create a solvent environment where the high nucleophilicity of MEG's primary hydroxyl groups is balanced by the moderated solvation and boiling point stability provided by DEG.^35,36^ Unlike traditional catalysts, DEA acts as a multi-functional kinetic promoter, facilitating the formation of intermediate complexes that lower the activation energy for urethane cleavage without significantly accelerating the secondary degradation routes to TDA.^37,38^

A core component of this study is the mapping of the multi-variable operational landscape—optimizing the glycol-to-PUF ratio, catalyst dosage, temperature, and residence time. We demonstrate that by operating within a tightly constrained “reaction window” at 160 °C, it is possible to maximize urethane scission while arresting the fragmentation of hard segments, thereby suppressing TDA formation to a record-low concentration of 2278 mg kg^−1^. To bridge the gap between chemical results and environmental safety, we integrate *in silico* toxicity estimation using the ECOSAR (96 h LC_50_) model. This regression-based approach allows for a transparent assessment of the aquatic toxicity of the resulting glycolysates, providing a quantitative metric for the safety of recycled polyols. Ultimately, this work provides a scalable mechanistic paradigm for the low-toxicity, high-quality chemical recycling of PUF waste, supporting a 40% loading of recycled content—far exceeding the current industry standard for safe incorporation.

## 2 Results and discussion

### 2.1 Mechanistic evaluation of binary-glycol synergism in PUF glycolysis

**Fig. 1:**
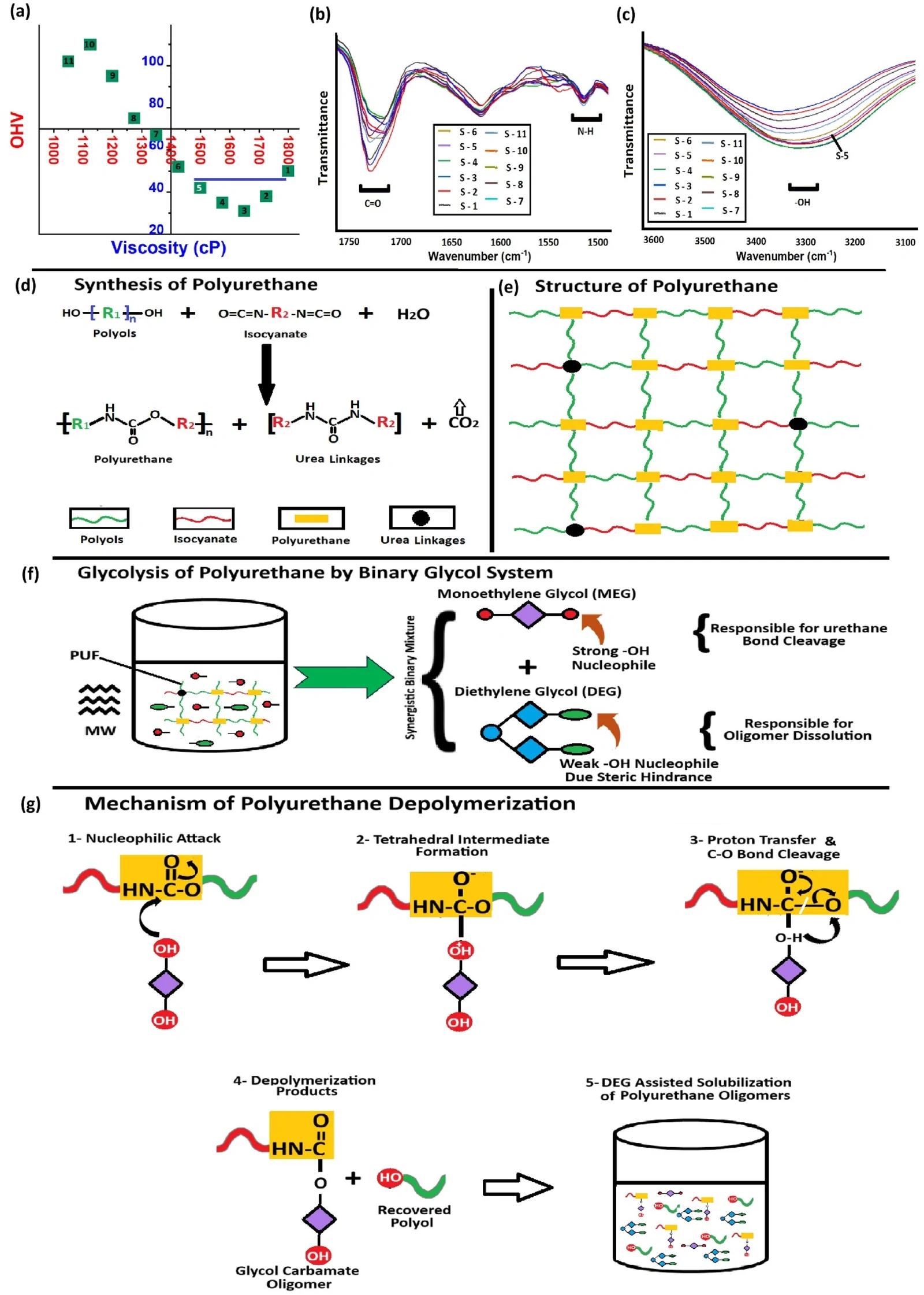
Mechanistic evaluation of the synergistic binary glycol system for polyurethane foam (PUF) depolymerization. (a) Hydroxyl value (OHV) *versus* viscosity for recovered polyol samples (S1–S11). (b and c) FTIR spectra highlighting the attenuation of urethane C=O and N–H bands (b) and the –OH stretching region (c). (d and e) Standard chemical synthesis route (d) and representative crosslinked structure (e) of virgin polyurethane. (f) Overview of the microwave-assisted (MW) binary glycolysis system, illustrating the decoupled roles of monoethylene glycol (MEG) and diethylene glycol (DEG). (g) Proposed reaction mechanism for PUF depolymerization, demonstrating MEG-driven nucleophilic cleavage followed by DEG-assisted oligomer solubilization.

To identify the optimal solvent environment for the chemoselective depolymerization of polyether-based PUF, we systematically evaluated eleven MEG–DEG formulations (S1–S11). As shown in Fig. 1a, the hydroxyl value (OHV)—representing the stoichiometric density of reactive nucleophiles—exhibits a linear monotonic decay from 1800 mg KOH g^−1^ (S1) to 1050 mg KOH g^−1^ (S11).^13^ Crucially, rheological analysis of the unreacted binary solvent mixtures reveals near-ideal physical mixing behavior (SI). The neat solvent viscosities decrease linearly from 45 cP (S1) to 36 cP (S11), yielding a small and slightly negative Grunberg–Nissan interaction parameter (*d* ≈ −0.096), as described by eqn (1).^39^

1 ln(*η*_mix_) = *x*_1_ ln(*η*_1_) + *x*_2_ ln(*η*_2_) + *x*_1_*x*_2_*d*

where *x* represents the mole fraction, *η* the dynamic viscosity, and *d* a constant proportional to the interchange energy. The small magnitude of *d* indicates weak non-ideal interactions but no significant structural perturbation of the liquid phase, confirming that the solvent system behaves as a predictable continuum prior to reaction. In contrast, the viscosity of the resulting glycolysates exhibits a pronounced non-linear response as a function of composition. Although the lowest viscosity is observed at S3, the overall trend follows a U-shaped profile, with substantially higher viscosities at both MEG-rich (S1) and DEG-rich (S11) extremes. This divergence from the near-ideal behavior of the initial solvent mixture indicates that the observed response is not inherited from solvent properties but rather emerges from reaction-induced structural evolution.

Importantly, formulation S5 (MEG : DEG = 6 : 4) is identified as the optimal system, not because it corresponds to the absolute viscosity minimum, but because it achieves a favorable balance between reduced MEG content and efficient depolymerization performance, yielding a significantly lower glycolysate viscosity than pure MEG (S1) while maintaining sufficient reactivity. This establishes that the optimal composition is governed by a kinetic-solvation trade-off, rather than a single extremum in viscosity. This behavior can be rationalized by considering the competing contributions of bond cleavage and fragment stabilization. At high MEG content, rapid urethane scission generates a high concentration of reactive intermediates, which can undergo secondary association, leading to increased viscosity. Conversely, at high DEG content, reduced nucleophilic activity limits depolymerization efficiency, resulting in larger residual fragments and higher viscosity. The intermediate compositions therefore represent a regime in which fragment generation, and solvation are optimally balanced, suppressing both incomplete depolymerization and post-cleavage aggregation.

### 2.2 Kinetic divergence and steric regulation

The origin of this emergent synergism lies in the decoupled mechanistic roles of MEG and DEG. The intrinsic reactivity of these glycols toward urethane bond cleavage can be rationalized using the Taft relationship (eqn (2)), which separates electronic and steric contributions to the reaction rate constant (*k*).^40^

2 log(*k*/*k*_0_) = *ρσ* + *δE*_s_

where *σ* and *E*_s_ are the polar and steric substituent constants, and *ρ* and *δ* represent their respective sensitivity coefficients. As depicted in the proposed mechanistic pathway (Fig. 1g), DEG exhibits a significantly more negative steric parameter *E*_s_ due to its extended chain length and internal ether linkage. This imposes structural constraints on the approach of the terminal hydroxyl group to the electrophilic urethane carbonyl, reducing the effective collision frequency and lowering the intrinsic rate of nucleophilic attack. Conversely, MEG carries a minimal steric penalty, allowing it to act as a potent nucleophile that facilitates highly efficient C–O bond scission (Fig. 1g, steps 1–4).^41^ However, kinetic superiority alone is insufficient to ensure optimal process performance. To investigate the origin of the proposed solvent-system synergy, FTIR spectra of pure MEG, pure DEG, and the optimized MEG : DEG (6 : 4) binary solvent have been compared (Fig. S1). The binary solvent exhibits subtle but reproducible differences in the O–H stretching envelope (3200–3600 cm^−1^) together with variations in the C–O stretching/fingerprint region relative to the individual glycols. These spectral changes are consistent with modified intermolecular hydrogen-bonding interactions within the mixed solvent, indicating that the binary solvent establishes a distinct local chemical environment prior to glycolysis. This direct experimental evidence supports the proposed solvent-system synergy which complements the kinetic and thermodynamic analyses. Further, FTIR analysis (Fig. 1b and c) reveals a progressive attenuation of the urethane carbonyl (∼1725 cm^−1^) and N–H bending (∼1530 cm^−1^) bands across the sample series. Intermediate compositions—particularly S5—exhibit robust suppression of these characteristic features. This trend confirms enhanced depolymerization under mixed-glycol conditions, demonstrating that an overwhelming excess of MEG is not requisite to achieve substantial bond cleavage. The complementary necessity of DEG becomes fully evident when evaluating macroscopic solvation effects. The Hansen solubility parameter distance (*R*_a_), calculated using eqn (3):^42^

3 (*R*_a_)^2^ = 4(*δ*_d1_ − *δ*_d2_)^2^ + (*δ*_p1_ − *δ*_p2_)^2^ + (*δ*_h1_ − *δ*_h2_)^2^

decreases progressively with increasing DEG content. This shift reflects improved thermodynamic compatibility between the solvent environment and the polyether segments of the degrading polymer matrix. At optimized intermediate compositions, this reduction in *R*_a_ enhances the dispersion of depolymerized fragments (Fig. 1, step 5) and actively suppresses intermolecular association, thereby limiting viscosity growth within the recovered phase (Fig. 1a). Collectively, these findings substantiate a degradation mechanism governed by kinetic–thermodynamic decoupling: MEG acts as the primary kinetic driver for targeted urethane bond cleavage, while DEG functions as a vital thermodynamic regulator that solubilizes and stabilizes the resulting oligomers.^43^ The optimal performance observed at S5 represents the ideal convergence of these phenomena—retaining sufficient nucleophilic reactivity while maximizing solvation to prevent fragment re-association. The combined FTIR, GPC, viscosity, ^1^H NMR (SI Fig S2), and HPLC results provide convergent support for the proposed kinetic-solvation interpretation. FTIR confirms progressive attenuation of urethane-related bands, while GPC and viscosity measurements demonstrate corresponding molecular-weight and rheological changes associated with depolymerization. The ^1^H NMR spectrum of the purified glycolysate further supports the predominance of glycol/polyether-derived product species, with only weak signals in the aromatic region. In parallel, HPLC provides specific quantification of TDA, the principal degradation product of environmental concern in this system. The combined results indicate that the 6 : 4 MEG : DEG composition provides a favorable balance between effective urethane cleavage and limitation of secondary degradation. Because the glycolysate comprises a complex mixture of depolymerization products, individual non-TDA products cannot be assigned and quantified reliably using the present analytical approach. Thus, the proposed pathway is considered a mechanistically supported interpretation of the observed selectivity rather than direct evidence of individual transient intermediates. This balanced synergy provides a definitive mechanistic basis for the selective recovery of high-quality, low-viscosity polyols, underscoring the critical importance of co-tuning reactivity and solvation in sustainable polyurethane recycling systems.

### 2.3 Process optimization and kinetic control of TDA suppression

The suppression of primary aromatic amines (PAAs) is the central challenge in polyurethane (PU) upcycling, as the presence of toluene diamine (TDA) dictates the regulatory safety and downstream mechanical performance of the recycled polyols. Utilizing the synergistic S-5 binary-glycol system, we conducted a systematic mapping of the multi-variable operational landscape to decouple the targeted urethane scission from secondary degradative pathways. The coupled effects of reaction time, thermal conditions, catalytic loading, and solvent stoichiometry were systematically investigated using FTIR spectroscopy, rheological analysis, gel permeation chromatography (GPC), and quantitative TDA detection. The underlying mechanistic pathways governing these interdependent variables are discussed in the following sections.

#### 2.3.1 Temporal evolution and the oligomer stability window

**Fig. 2:**
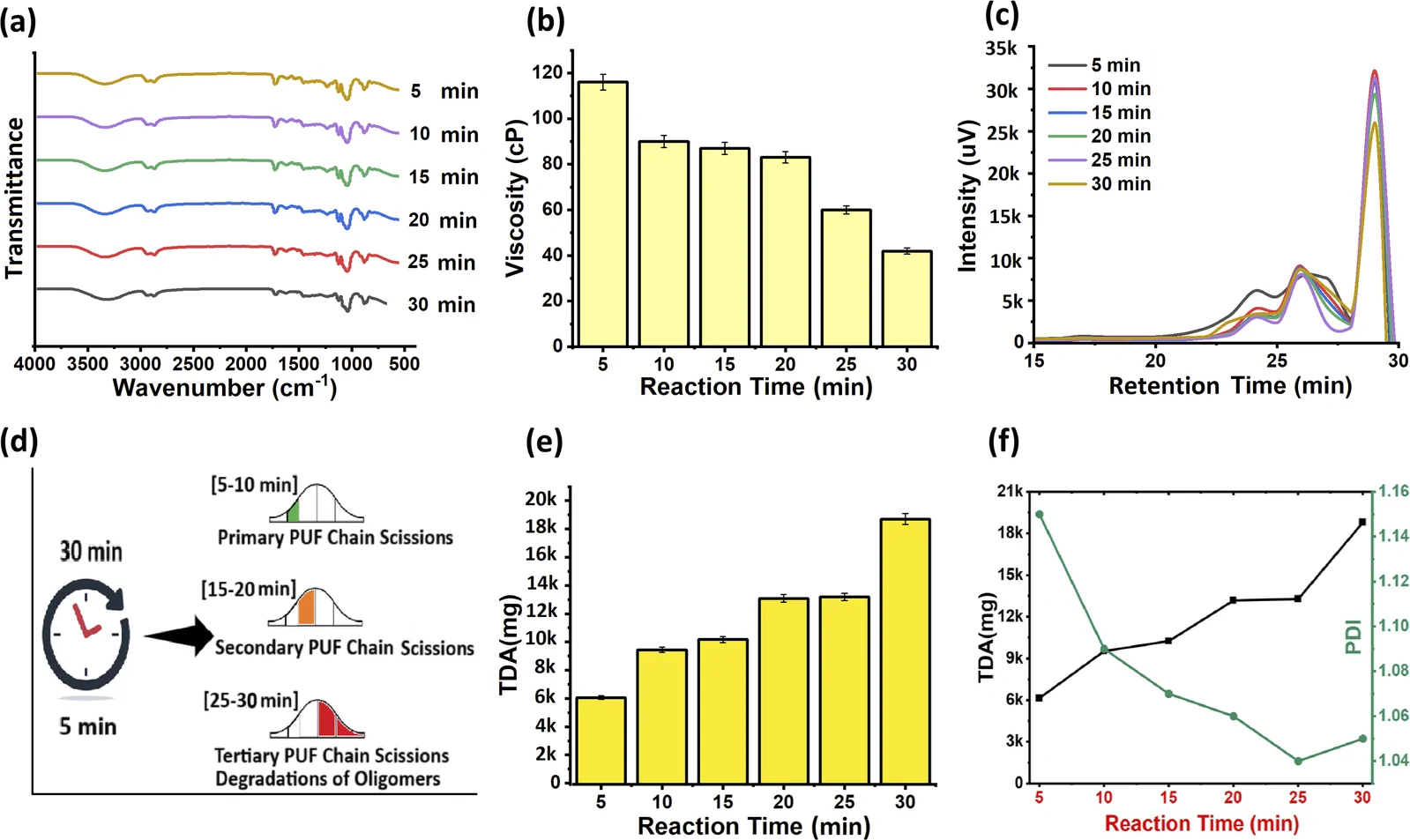
Kinetically bounded window for selective depolymerization and TDA suppression: (a–c) Time-resolved evolution of chemical structure, macro-rheology, and molecular weight distributions. (d–f) Mechanistic pathway identifying the “oligomer stability window” (10–20 min) that minimizes toxic aromatic amine release.

The coupled evolution of chemical structure, rheology, and molecular weight distributions in Fig. 2a–f defines a kinetically bounded depolymerization pathway governed by the formation and depletion of oligomeric intermediates. In the early stage (≈5–10 min), incomplete attenuation of urethane bands in Fig. 2a, together with high viscosity in Fig. 2b and GPC distributions skewed toward high molecular weight species in Fig. 2c, indicate diffusion-limited transesterification within a partially intact network. As illustrated in Fig. 2d, this regime is dominated by primary PUF chain scissions, where restricted mass transport limits effective glycol access to the polymer backbone. Consistently, reaction times below 30 min yield incomplete liquefaction, underscoring the need to overcome these transport constraints for full phase homogenization.^29^

A sharp transition emerges at ≈10–20 min, where the viscosity drop in Fig. 2b coincides with a pronounced shift in Fig. 2c toward lower molecular weights and a decrease in polydispersity index (PDI) shown in Fig. 2f. This regime defines the oligomer stability window, characterized by secondary chain scissions (Fig. 2d) in which urethane bond scission (Fig. 2a) proceeds efficiently while maintaining a stabilized population of soluble oligomers. Critically, Fig. 2e and f show that TDA formation remains relatively suppressed within this window, demonstrating that transesterification kinetically outcompetes thermolytic degradation. The persistence of oligomeric species (Fig. 2c) functions as a kinetic buffer, dissipating thermal energy and limiting the exposure of urea linkages to conditions that promote decarboxylation and amine formation.^37,44,45^

Beyond ≈20 min, the progressive depletion of this oligomer pool is evident from the continued leftward shift and narrowing of GPC traces (Fig. 2c), accompanied by low viscosity (Fig. 2b) and a sharp, non-linear increase in TDA concentration (Fig. 2e and f). The plateauing of FTIR changes in Fig. 2a indicates that primary urethane cleavage is largely complete; however, further residence time drives tertiary degradation of oligomers (Fig. 2d). Thus, although a 30 min reaction time ensures complete liquefaction, it lies at the upper boundary of the selectivity window, where additional processing disproportionately accelerates urea decomposition.^45^ Collectively, Fig. 2a–f demonstrates that selective polyurethane glycolysis is intrinsically time-resolved: optimal depolymerization and minimal toxic byproduct formation occur within a transient kinetic window defined by oligomer persistence. Precise temporal control is therefore essential to maximize circularity while suppressing degradation.

#### 2.3.2 Thermal regime and the selectivity–severity trade-off

**Fig. 3:**
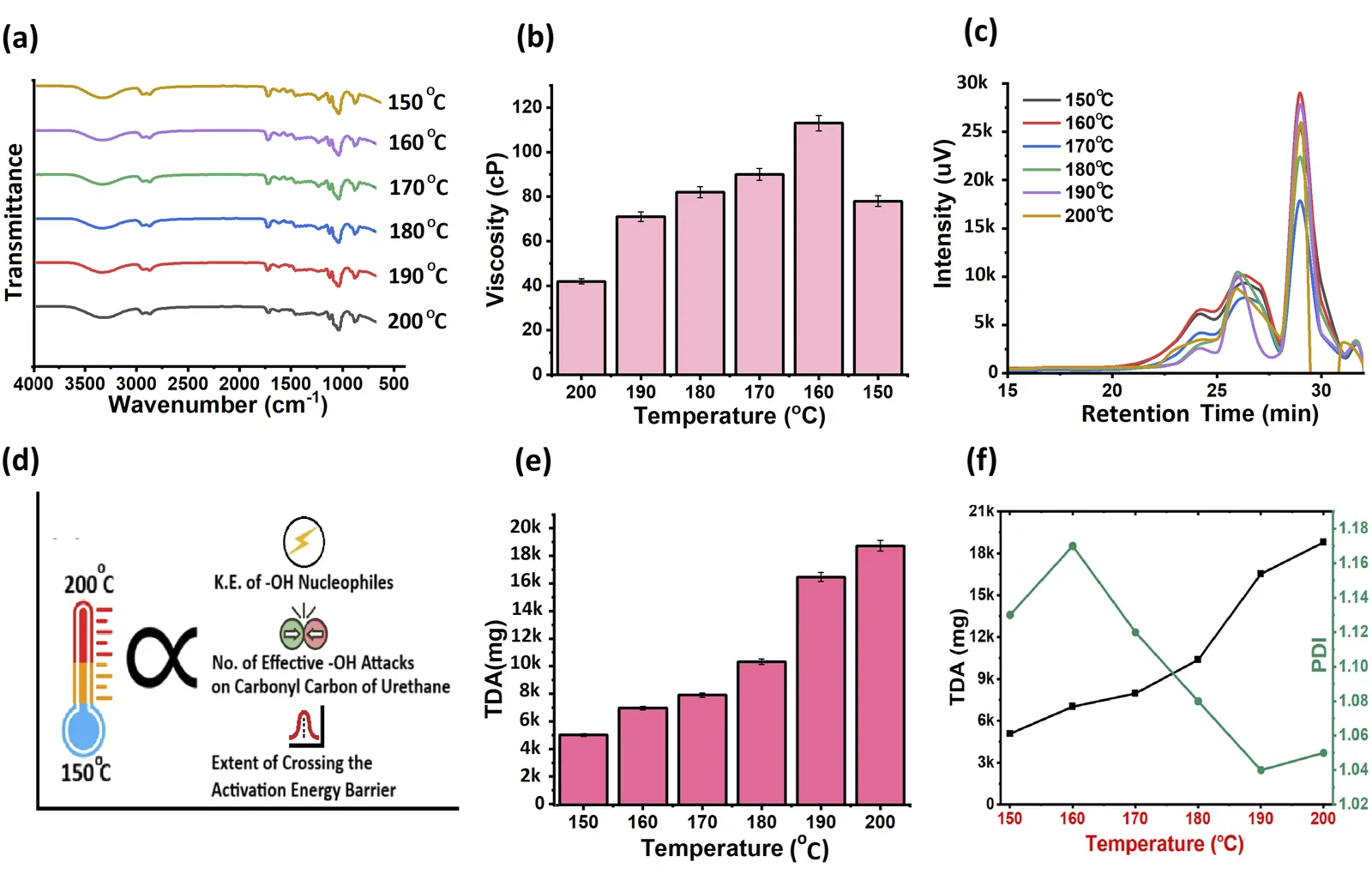
Thermal regulation and the selectivity–severity trade-off. (a–c) Temperature-dependent structural transitions and molecular weight profiles. (d–f) Mechanistic activation and exponential TDA growth identifying the 160–170 °C threshold for maintaining chemoselectivity.

The temperature-dependent behavior illustrated in Fig. 3a–f reveals a tightly coupled selectivity–severity trade-off, where increasing thermal input enhances urethane cleavage but accelerates secondary degradation. FTIR spectra (Fig. 3a) confirm progressively greater urethane bond dissociation with rising temperatures, evidenced by the attenuation of characteristic carbamate vibrations. Interestingly, the viscosity profile (Fig. 3b) exhibits a non-monotonic trend, reaching a maximum at 160 °C. This peak likely corresponds to a transition state of maximum oligomeric complexity or initial polymer swelling before significant chain scission dominates at higher temperatures 170 °C, where viscosity drops to a minimum of 42 cP at 200 °C. GPC profiles (Fig. 3c) further corroborate this, showing a transition from broad, oligomer-rich distributions at 150–160 °C to sharp, low-molecular-weight peaks at ≥190 °C. This shift reflects a move from kinetically controlled transesterification toward aggressive, thermally driven over-fragmentation. Mechanistically (Fig. 3d), while moderate temperatures provide the activation energy necessary for –OH nucleophilic attack on urethane carbonyls, higher thermal regimes disproportionately activate deleterious urea cleavage and decarboxylation pathways.^14,46^

This transition is quantitatively captured by the exponential increase in toluenediamine (TDA) concentration (Fig. 3e) and the concurrent fluctuations in the Polydispersity Index (PDI) (Fig. 3f). The sharp decline in PDI above 160 °C indicates that elevated temperatures promote a “leveling off” of molecular weight through non-selective degradation into basic aromatic units. While 200 °C achieves the lowest viscosity, it results in a four-fold increase in toxic TDA compared to 150 °C. Consequently, the ∼160–170 °C window represents the critical “tipping point” where oligomeric diversity is still preserved before the onset of rapid aromatic amine formation. Precise thermal regulation is therefore paramount to maintain chemoselectivity, aligning with recent kinetic models of polyurethane glycolysis.^28^

#### 2.3.3 Catalytic density and active site accessibility

**Fig. 4:**
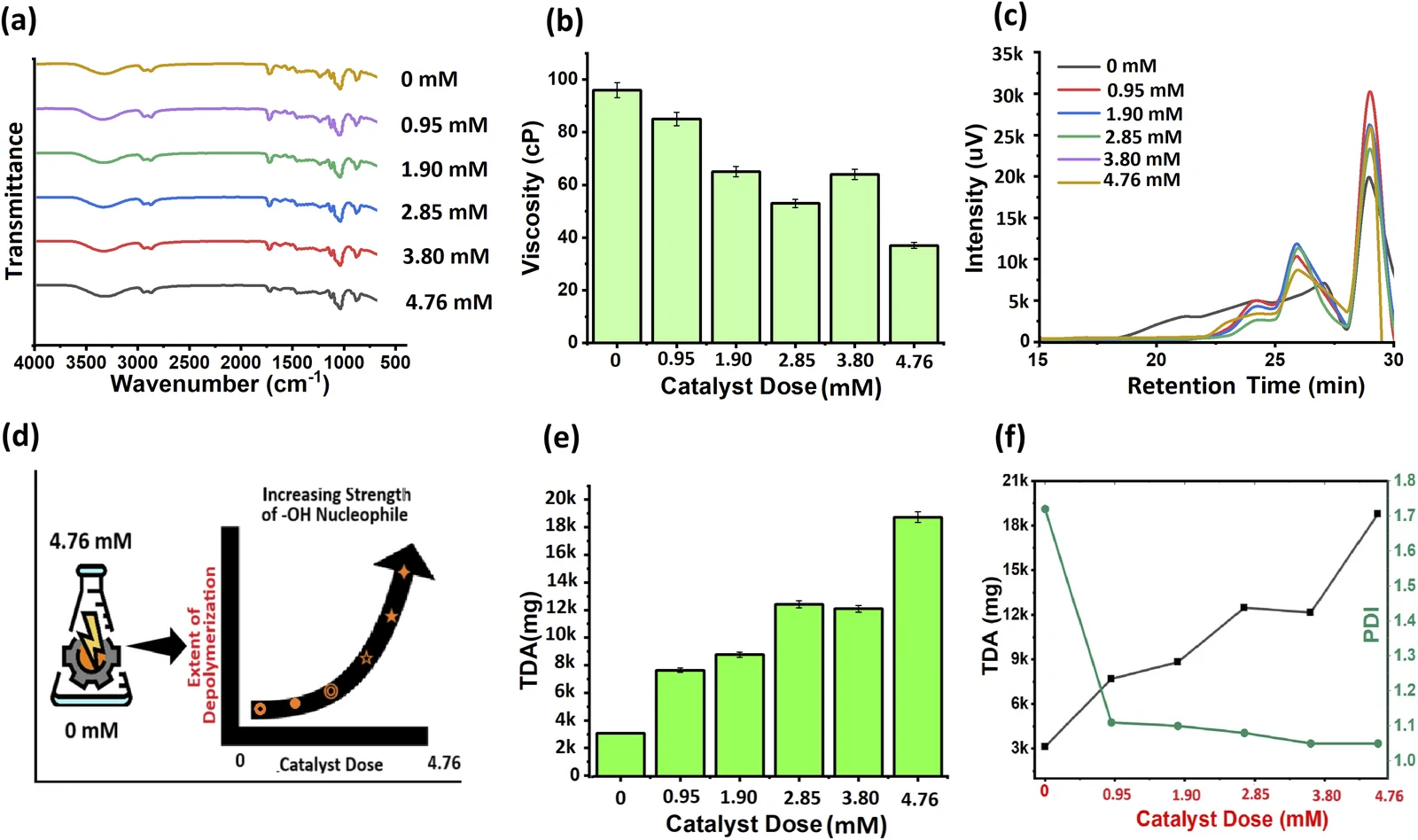
Impact of catalytic density on active site accessibility and byproduct formation. (a–c) Influence of DEA loading on chain scission kinetics and viscosity. (d–f) Modeling the transition from controlled depolymerization to non-selective degradation and “active site overcrowding” at high catalytic densities.

The catalytic efficiency of diethanolamine (DEA) in polyurethane (PU) depolymerization follows a non-linear trajectory governed by the interplay between nucleophilic potency and mass-transport limitations within the evolving polymer matrix. As illustrated in Fig. 4a–c, the introduction of DEA initiates a rapid reduction in polymer integrity. FTIR spectra (Fig. 4A) reveal a progressive attenuation of the urethane carbonyl stretching and an increase in hydroxyl signatures, correlating with the sharp decline in macro-scale viscosity from ∼98 cP to ∼38 cP (Fig. 4b). It is noteworthy that the viscosity of the reaction mixture at 3.80 mM DEA is slightly higher than that at 2.85 mM. This non-monotonic behavior may arise from the competing processes of polyurethane chain cleavage, oligomer formation, and subsequent degradation during glycolysis. At 2.85 mM DEA, sufficient urethane bond cleavage occurs to generate relatively low-molecular-weight soluble products, resulting in lower viscosity. At the higher DEA concentration of 3.80 mM, accelerated degradation of the polyurethane network may initially increase the concentration of partially depolymerized oligomeric species and strengthen intermolecular interactions within the glycolysate, leading to a temporary increase in viscosity. The GPC results (Fig. 4c) and the conforming TDA concentration at higher DEA loading (Fig. 4e) further indicate that increasing catalyst concentration alters the molecular-weight distribution and promotes secondary degradation. Therefore, the observed viscosity increase at 3.80 mM DEA likely reflects the dynamic balance between oligomer formation and subsequent chain degradation rather than a simple dependence of viscosity on catalyst loading. This macro-rheological change is corroborated by Gel Permeation Chromatography (GPC) profiles (Fig. 4c), where increasing catalyst loading shifts the molecular weight distribution toward higher retention times (25–30 min), signifying the cleavage of long-chain carbamates into smaller oligomeric fragments. The mechanism is driven by DEA's bifunctional nature: the hydroxyl groups facilitate transesterification while the secondary amine acts as a proton-shuttle, lowering the Gibbs free energy of activation for nucleophilic attack on the urethane carbonyl.^28,44^ However, the data suggests a critical threshold in catalytic density. While depolymerization extent (represented by viscosity reduction) plateaus or fluctuates beyond ∼2.85 mM (Fig. 4b), the formation of 2,4-toluenediamine (TDA)—a byproduct of complete urethane cleavage—continues to rise sharply, reaching ∼19 k mg at 4.76 mM (Fig. 4e and f).

This divergence, modeled in Fig. 4d, indicates a transition from controlled depolymerization to non-selective degradation. At low concentrations (<1 mM), the Polydispersity Index (PDI) drops precipitously from 1.7 to ∼1.1 (Fig. 4f), suggesting a highly selective “chain-end” or “uniform cleavage” mechanism. Beyond this, the PDI stabilizes even as TDA concentration surges. This implies that excessive local concentrations of –OH/–NH sites do not further improve polymer breakdown efficiency but instead accelerate secondary aminolysis and urea cleavage pathways.^2,47^ Mechanistically, this behavior suggests that at high loadings, the system becomes limited by “active site overcrowding,” where catalyst–catalyst interactions or local saturation within the viscous microenvironment favor the formation of small-molecule aromatic amines over productive polymer chain scission. Thus, optimal chemoselectivity is achieved not through maximum loading, but by balancing nucleophilic strength with the spatial accessibility of the urethane linkages within the solvated network.

#### 2.3.4 Solvent stoichiometry and solvation dynamics

**Fig. 5:**
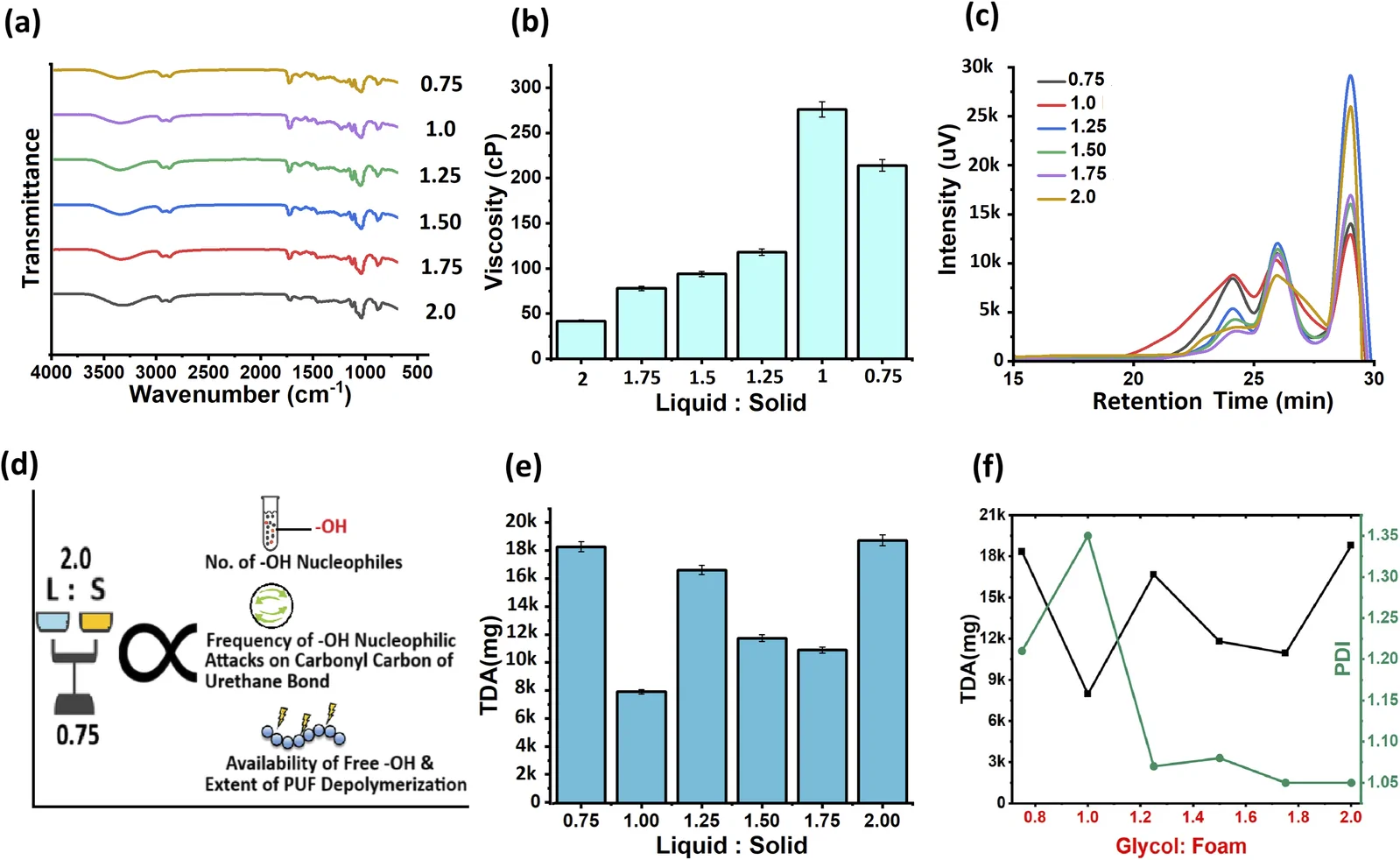
Influence of solvent stoichiometry and solvation dynamics on reaction uniformity. (a–c) Effect of liquid-to-solid (L : S) ratio on transport and material homogeneity. (d–f) Mechanistic schematic of transport-limited *versus* nucleophile-dense regimes and their impact on toxic byproduct formation.

The depolymerization of polyurethane (PU) *via* glycolysis is fundamentally governed by a competitive coupling between nucleophilic flux and viscoelastic impedance. As evidenced by the rheological and gravimetric data inFig. 5b and e, the liquid-to-solid (L : S) ratio acts as the primary modulator of the medium's transport properties. At the critical stoichiometry of L : S = 1.0, the system encounters a pronounced viscosity peak (∼275 cP), creating a transport-limited bottleneck. In this regime, the dense concentration of partially solvated oligomeric fragments restricts the effective collision frequency of the catalyst. This diffusion-limited state is quantitatively characterized by a local minimum in the toluene diamine (TDA) and a corresponding maximum in the Polydispersity Index (PDI) of ∼1.34 (Fig. 5f), indicating a highly heterogeneous reaction environment where chain scission is non-uniform. Interestingly, the system exhibits a “rebound” effect at the sub-stoichiometric ratio of 0.75. Despite a relatively high macroscopic viscosity (Fig. 5b), the TDA increases significantly (Fig. 5e). This suggests a regime dominated by nucleophilic density, where the high spatial proximity of –OH groups to the urethane carbonyls—illustrated in the mechanistic schematic in Fig. 5d—compensates for restricted molecular mobility.^48^ FTIR spectra (Fig. 5a) support this, showing characteristic carbamate vibrations and hydroxyl stretching intensities that correlate with the extent of primary scission.^7^

Transitioning to solvent-rich conditions (L : S > 1.5) shifts the system into a solvokinetic-optimized regime. The resulting order-of-magnitude reduction in viscosity to below 100 cP (Fig. 5b) effectively eliminates the mass transfer barriers identified in concentrated melts, allowing for higher catalyst turnover frequencies.^49^ This rheological thinning promotes a transition toward thermodynamic equilibrium, reflected in the stabilization of the PDI at a near-monodisperse value of ∼1.05 and the convergence of GPC retention times toward the 26–29 min window (Fig. 5c and f). By balancing the frequency of nucleophilic attack with the requirements of mass transfer fluidity, an L : S ratio between 1.5 and 2.0 emerges as the optimal window for producing uniform secondary polyols, ensuring the chemical homogeneity required for high-fidelity closed-loop manufacturing.^14^

### 2.4 Integrated process optimization and toxicological assessment

Integrating the full dataset identifies a narrow operational window in which urethane bonds are fully cleaved while secondary decomposition pathways remain suppressed. Reaction conditions defined by temperatures of 150–170 °C, catalyst loadings near 1 mM, and a 1 : 1 glycol : PUF ratio provide the optimal balance between depolymerization efficiency and chemical selectivity. Under these specific optimized settings—160 °C for 30 minutes—the toluenediamine (TDA) formation decreased to 2278 mg, the lowest value observed across the experimental matrix. To further characterize the molecular composition of the recovered product, the purified glycolysate obtained under the optimized reaction conditions was analyzed by ^1^H NMR spectroscopy (SI Fig. S2). The spectrum is dominated by resonances in the *δ* ∼3.3–4.3 ppm region, characteristic of protons in oxygenated aliphatic environments (*e.g.*, –CH_2_–O–) and consistent with the glycol/polyether-rich nature of the recovered product. Additional lower-intensity resonances are observed in the downfield region, including weak signals within the aromatic proton region (approximately *δ* ∼6.5–8.0 ppm), indicating the presence of a minor aromatic fraction in the glycolysate. Given the low intensity and partial overlap of these resonances, their assignment to a specific aromatic species, including TDA, cannot be made unambiguously from the ^1^H NMR spectrum alone. Accordingly, ^1^H NMR was used as complementary qualitative evidence of the molecular composition of the recovered glycolysate, whereas reversed-phase HPLC was employed for specific quantitative determination of TDA. The optimized glycolysate contained 2278 mg kg^−1^ TDA, as quantified using 2,4- and 2,6-diaminotoluene calibration standards. Taken together, the ^1^H NMR and HPLC results indicate that the recovered product is predominantly composed of glycol/polyether-derived species, with a comparatively minor but measurable aromatic-amine fraction under the optimized glycolysis conditions.

These findings demonstrate that controlling reaction severity, rather than simply maximizing the depolymerization rate, is essential for producing high-quality, low-toxicity glycolysates. When operated within this engineered window, the inherent synergy of the solvent system enables efficient polyurethane (PU) depolymerization while significantly suppressing the formation of hazardous aromatic amines.

**Fig. 6:**
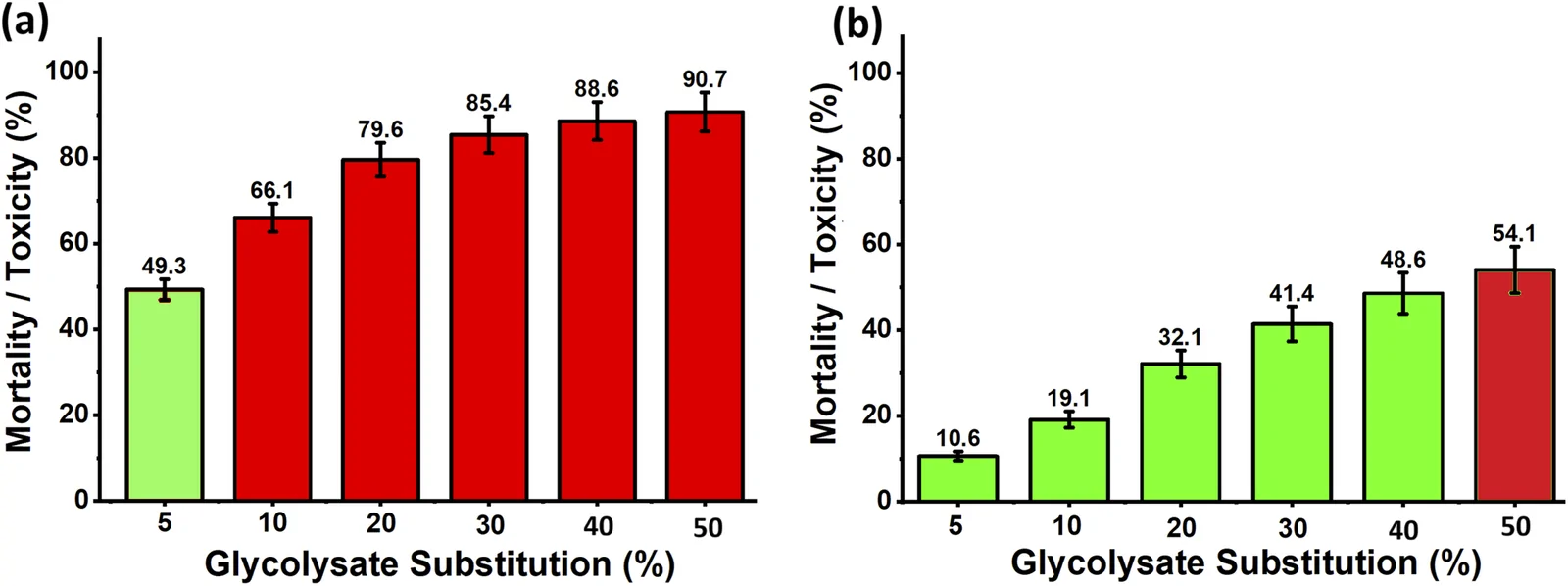
Toxicological assessment and feasibility of circular substitution. (a and b) Predicted aquatic mortality profiles for non-optimal *versus* optimized glycolysis streams, demonstrating the expanded safety window for high-content (40%) material substitution.

The predicted acute toxicity profile underscores a strong dependence of aquatic mortality on the TDA content introduced *via* glycolysate substitution in recycled PU formulations. As shown in Fig. 6a, the non-optimal stream generates TDA concentrations that exceed the ECOSAR-derived LC50 (lethal concentration for 50% of the population) even at low substitution levels. This results in consistently high predicted mortality—exceeding 66%—for all formulations where substitution is 10% or higher. This trend illustrates the disproportionately elevated TDA burden associated with non-optimized glycolysis routes and indicates that meaningful material substitution cannot be achieved in these streams without violating acute toxicity thresholds. In contrast, the optimal glycolysis stream shown in Fig. 6b yields a markedly reduced toxicological footprint. Substitutions up to 40% remain below the critical LC50 boundary, producing mortality levels at or below 48.6%. Only at the 50% substitution level does the mortality cross the threshold to 54.1%. This clear divergence underscores the critical role of process optimization in minimizing TDA, thereby expanding the feasible substitution window for recycled feedstocks. Ultimately, these results highlight that chemical-process improvements, rather than formulation adjustments alone, are the essential lever for achieving environmentally compatible circularity in polyurethane recycling.

To benchmark the proposed methodology, the performance of the present DEA-catalyzed binary glycol system was compared with representative catalytic systems reported for polyurethane glycolysis Table 1. Although alkaline, metal-based, and tertiary amine catalysts can promote efficient depolymerization, they may require higher catalyst loadings, elevated temperatures, and/or longer reaction times, which can increase secondary degradation and undesired aromatic amine formation. In contrast, the binary MEG solvent system enables efficient depolymerization under comparatively mild conditions while substantially suppressing toluenediamine (TDA) formation. This enhanced performance is attributed to the synergistic catalyst–solvent interaction, which facilitates urethane bond cleavage while promoting effective solvation of the depolymerization products. Overall, the results highlight solvent engineering as an effective strategy for improving chemoselectivity and product quality during polyurethane chemical recycling.

**Table 1:** Comparison of reported polyurethane glycolysis catalysts with the present DEA/MEG : DEG system for TDA suppression

Catalyst	Solvent	Temperature (°C)	Catalyst loading	Main outcome	Ref.
DEA (this work)	MEG : DEG (6 : 4)	160	1 mM	Low TDA formation, high-quality glycolysate, and chemoselective depolymerization	This work
DABCO	DEG	200	0.1 wt%	Efficient glycolysis; high temperature, TDA suppression not assessed	50
1. Titanium *n*-butoxide	DEG	189	Not reported	Effective glycolysis but split phase product; high temperature, TDA control not established	33
2. Octoate salt					
Tin(ii) octoate [Sn(Oct)_2_]	DEG	190	Not reported	High activity; TDA control not established	51
KOH	GPX-600/3000/6000	195	1.0 wt%	Effective glycolysis; TDA control not established	52
NaOH	Glycerol	160–220	Not reported	Effective cleavage; TDA suppression not assessed	53
Potassium acetate (KAc)	DEG	220	1 wt% of PU	High PU conversion; higher amine formation reported, limiting TDA/amine selectivity	54
Dibutyltin dilaurate (DBTDL)	Glycerin	220	0.5 wt%	High glycolysis efficiency and selectivity; high reaction temperature and metal-containing catalyst	55
Zn/Sn/Al hydrotalcite (HTC)	DEG	190	0.01 g	Effective heterogeneous catalysis and high polyol recovery; relatively high temperature and heterogeneous catalyst separation required	56

## 3 Conclusion

In summary, we have demonstrated that the long-standing challenge of toxic byproduct formation in polyurethane (PU) glycolysis can be resolved through a synergistic, solvent-engineered approach. By transitioning from conventional single-solvent media to a precisely tuned 6 : 4 MEG : DEG binary system, we established a chemoselective pathway that prioritizes primary urethane transesterification while arresting the thermolytic pathways leading to aromatic amine generation. This solvent synergy is rooted in a fundamental kinetic–thermodynamic balance: MEG provides the high nucleophilic density required for rapid chain scission, while DEG facilitates the stabilization of emergent oligomers and mitigates mass-transport limitations.

Our systematic mapping of the multivariable operational space identifies a critical reaction manifold—defined by a 1 : 1 glycol-to-PUF ratio and a 160 °C thermal threshold—that minimizes the toluenediamine (TDA) burden to a record-low 2278 mg kg^−1^. Crucially, by integrating ECOSAR-based toxicological modeling with materials performance data, we provide evidence that this refined process directly translates to industrial feasibility. The resulting glycolysate enables up to 40% substitution in secondary PU formulations while strictly adhering to acute aquatic safety standards, a threshold previously unattainable with high-severity chemical recycling. Ultimately, this work provides a scalable and mechanistically grounded blueprint for the upcycling of complex thermoset polymers. By resolving the “selectivity–severity” paradox through molecular-level solvent design, we offer a path forward for the commercially viable and environmentally compliant production of recycled materials, bridging the gap between laboratory-scale chemical recycling and a truly circular plastics economy.

## 4 Methods

### 4.1 Materials

Scrap flexible polyurethane (PU) foam waste obtained from automotive seating (low-density, polyether-based) was used as the primary feedstock; it consists predominantly of toluene diisocyanate (TDI)-derived hard segments and trifunctional high-molecular-weight polyether polyols. Monoethylene glycol (MEG, ≥99%) and diethylene glycol (DEG, ≥99%) were employed as glycolysis agents. Diethanolamine (DEA) was employed as catalyst in glycolysis. Analytical-grade methanol, tetrahydrofuran (THF, HPLC grade), and potassium hydroxide (KOH) were used for sample preparation and as eluents in gel permeation chromatography (GPC) and high-performance liquid chromatography (HPLC), while 2,4-diaminotoluene and 2,6-diaminotoluene served as external calibration standards. Hydroxyl value (OHV) was determined using *N*-methyl-2-pyrrolidone, acetic anhydride, and 4-dimethylaminopyridine (DMAP) *via* acetylation. All reagents were of analytical grade and used as received unless otherwise specified.

### 4.2 Methods

#### 4.2.1 Glycolysis of PUF

Flexible polyurethane foam (PUF) waste obtained from post-consumer furniture was mechanically milled using a ZM 300 centrifugal mill (Retsch, Germany) to obtain particles with an average size of approximately 1 mm. The milled PUF was subsequently dried prior to glycolysis to remove residual moisture and ensure consistent reaction conditions. Unless otherwise specified, the dried PUF was used directly for the glycolysis experiments. Glycolysis experiments were performed using a MicroSYNTH microwave reactor (Milestone S.r.l., Sorisole, Italy) equipped with a magnetic stirring system, a fiber-optic temperature probe, and hermetically sealed 100 mL PTFE reaction vessels. The fiber-optic probe was used for continuous monitoring of the reaction temperature during microwave irradiation. In a typical experiment, 9.0 g of dried PUF was combined with the glycolytic solvent at a glycol-to-PUF mass ratio of 2 : 1. Monoethylene glycol (MEG), diethylene glycol (DEG), and their binary mixtures were investigated as glycolytic media. Diethanolamine (DEA) was used as the catalyst. Unless otherwise specified, the glycolysis reaction was conducted at 200 °C for 30 min under microwave irradiation with continuous magnetic stirring. The reaction temperature was monitored using the fiber-optic probe throughout the reaction. The sealed reaction vessel was allowed to cool after completion of the prescribed reaction time before opening and collecting the reaction mixture. Systematic experiments were subsequently performed to investigate the effects of reaction temperature, reaction time, DEA catalyst loading, glycol-to-PUF ratio, and glycol composition on the extent of PUF depolymerization and product characteristics. Particular attention was given to the formation of aromatic amines, especially toluenediamine (TDA), as an indicator of undesired secondary degradation during glycolysis.

#### 4.2.2 Polyol characterization

The recovered polyols obtained after polyurethane glycolysis were characterized using complementary analytical techniques to evaluate their chemical structure, hydroxyl functionality, rheological properties, molecular-weight distribution, and aromatic amine content. Prior to characterization, the recovered products were appropriately separated from insoluble residues and prepared according to the requirements of each analytical method.

Fourier-transform infrared spectroscopy (FTIR) was employed to qualitatively evaluate the chemical structure of the recovered polyols and to monitor changes in the characteristic functional groups associated with polyurethane depolymerization. Particular attention was given to the characteristic absorption bands associated with urethane groups, hydroxyl groups, and aromatic amine functionalities. Comparison of the spectra before and after glycolysis was used to assess changes in the chemical structure resulting from urethane bond cleavage. FTIR measurements were performed using a Nicolet iS50 FTIR spectrometer (Thermo Fisher Scientific, Waltham, MA, USA) equipped with a diamond crystal attenuated total reflectance (ATR) accessory. The ATR crystal was cleaned between measurements to minimize cross-contamination between samples. The recovered polyol samples were analyzed directly using the ATR configuration, avoiding the need for additional sample preparation. Spectra were collected over the wavenumber range of 4000–720 cm^−1^. The resulting spectra were evaluated by identifying characteristic absorption bands and comparing their relative changes among the PUF feedstock, glycolysis products, and recovered polyols. The hydroxyl value (OHV) of the glycolysis products and recovered polyols was determined according to ASTM D4274-11, using a Mettler Toledo G20S compact titrator (Columbus, OH, USA). The dynamic viscosity of the glycolysis products and recovered polyols was measured using a cone-and-plate viscometer (ATAC NµLine, Analytical Technology & Control Ltd, Devizes, UK) in accordance with ASTM D4878-15. Measurements were conducted at 25.0 ± 0.5 °C to ensure consistent comparison among samples. Samples were allowed to reach the specified measurement temperature before analysis, and care was taken to ensure homogeneous sample loading and the absence of visible particulate matter that could interfere with the measurement. Viscosity was used as an important indicator of the molecular characteristics and flow behavior of the glycolysates and recovered polyols.

The hydroxyl value (OHV) of the glycolysis products and recovered polyols was determined according to ASTM D4274-11. OHV measurements were performed using a Mettler Toledo G20S compact titrator (Columbus, OH, USA). The hydroxyl value was determined by the prescribed titrimetric procedure and expressed as the amount of potassium hydroxide equivalent to the hydroxyl functionality present in the sample. The OHV was used to evaluate changes in hydroxyl functionality resulting from polyurethane depolymerization and to assess the suitability of the recovered polyols for potential subsequent polyurethane synthesis.

Gel permeation chromatography (GPC) was employed to determine the molecular-weight distribution of the recovered polyols and to evaluate changes in molecular size resulting from polyurethane depolymerization. Prior to GPC analysis, the recovered polyols were separated from the glycolysis mixture and prepared as homogeneous solutions suitable for chromatographic analysis. GPC analyses were conducted using a JASCO PU-980 pump system (JASCO, Easton, MD, USA) equipped with three Phenogel size-exclusion columns (Phenomenex, Torrance, CA, USA) with a particle size of 5 µm and nominal pore sizes of 1000, 100, and 50 Å, respectively. A JASCO RI-830 refractive index detector was used for detection. The recovered polyol samples were dissolved in tetrahydrofuran (THF) at a concentration of 0.04 wt%. The resulting solutions were thoroughly homogenized and passed through 0.1 µm PTFE membrane filters prior to injection to remove insoluble particles and minimize the possibility of column blockage. THF was used as the mobile phase, and chromatographic separation was conducted at 40 °C with a flow rate of 0.35 mL min^−1^. The GPC results were used in conjunction with viscosity measurements to evaluate the relationship between molecular-weight distribution and the macroscopic properties of the recovered glycolysates.

^1^H NMR spectroscopy was performed on the purified glycolysate obtained under the optimized glycolysis conditions to provide complementary molecular-level characterization. The sample was prepared in a suitable deuterated solvent and analyzed at room temperature. Chemical shifts were referenced to the residual solvent signal. The spectrum was evaluated for the characteristic proton environments of the glycol/polyether-derived product phase and for any resolvable resonances attributable to aromatic amines, particularly TDA. Because the recovered glycolysate did not exhibit adequate miscibility with suitable deuterated solvents for preparation of a homogeneous solution, ^13^C NMR analysis was not considered reliable for this sample. Consequently, ^1^H NMR was used for qualitative structural characterization, whereas quantitative determination of TDA was performed independently by reversed-phase HPLC.

Aromatic amine content was quantified by reversed-phase high-performance liquid chromatography (HPLC) using a Waters e2695 separation module coupled with a Waters 2489 UV detector (Waters Corporation, Milford, MA, USA) operating at dual wavelength detection. Separation was performed on a C18 column with hydrophilic endcapping (4.6 × 250 mm, 5 µm, 300 Å pore size). The mobile phase consisted of Milli-Q water containing 0.05% trifluoroacetic acid (A) and HPLC-grade methanol (B), starting at 97% A with a linear gradient of 1% min^−1^ for 10 min. For analysis, a known mass of recovered polyol was dissolved in 0.1 M potassium hydroxide in methanol to account for the amphoteric nature of amines. The solution was sonicated, filtered through a 0.2 µm PTFE membrane, and 10 µL was injected into the HPLC system.

#### 4.2.3 Extent of PUF depolymerization and determination of polydispersity index

The extent of flexible polyurethane foam (PUF) depolymerization was quantified gravimetrically following glycolysis. In a typical experiment, 9 g of PUF was subjected to depolymerization under the specified reaction conditions. Upon completion, the reaction mixture was treated with acetone to selectively dissolve the glycolyzed products, while leaving behind any non-depolymerized solid residue. The insoluble fraction was separated by filtration using a Whatman filter paper (6 µm pore size), thoroughly washed with acetone to remove residual soluble species, and subsequently dried to constant weight. The mass of the recovered solid residue was used to evaluate the depolymerization efficiency according to eqn (4).4
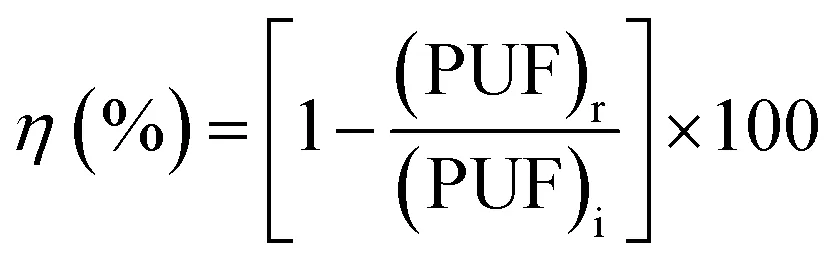
where *η* represents the depolymerization efficiency (%), (PUF)_r_ is the mass of residual, non-depolymerized foam (g), and (PUF)_i_ is the initial mass of PUF subjected to glycolysis (g).

The molar mass distribution and polydispersity index (PDI) of the glycolysis products were determined by gel permeation chromatography (GPC). The analyses were performed using a JASCO chromatographic system equipped with appropriate columns for polymeric and oligomeric species separation. Chromatographic data acquisition, peak integration, and molecular weight distribution analysis were conducted using ChromNAV (Version 2, JASCO Corporation) in conjunction with Spectral Manager (Version 2.5). Peak assignment and baseline correction were performed using automated algorithms embedded in the software, followed by manual verification to ensure accuracy. The number-average molecular weight (*M*_n_) and weight-average molecular weight (*M*_w_) were calculated from the obtained chromatograms based on standard calibration curves. The polydispersity index (PDI) was subsequently determined as the ratio *M*_w_/*M*_n_. All measurements were performed at least in duplicate to ensure reproducibility, and the reported values represent the average of independent analyses (SI).

#### 4.2.4 *In silico* toxicity assessment using ECOSAR

Acute aquatic toxicity was estimated using the ECOSAR v2.2 model implemented within EPI Suite 4.11. The neutral molecular structure of toluene-2,4-diamine (SMILES: Nc1ccc(N)cc1C) was used as input. To improve descriptor reliability, the experimentally determined octanol–water partition coefficient (log *K*_ow_ = 0.14) was provided, in agreement with the model-estimated value (0.16). Based on its structure, the compound was assigned to the amino–meta-aniline class within ECOSAR, and the corresponding quantitative structure–activity relationship (QSAR) for 96 h fish acute toxicity (LC_50_) was applied. Model outputs, including predicted physicochemical properties, regression parameters, and applicability domain diagnostics, were systematically recorded and evaluated. To assess dilution-dependent toxicity in both optimized and non-optimized glycolysates, the measured concentration of TDA was converted into effective concentrations (*C*_e_) at glycolysate substitution levels of 5, 10, 20, 30, 40, and 50%, according to eqn (5).

5 *C*_e_ = *C*_TDA,measured_ × *f*_dilution_

where *f*_dilution_ represents the volumetric fraction of glycolysate in the aqueous phase. The resulting *C*_e_ values were compared with the ECOSAR-predicted LC_50_ to calculate toxic units (TU), defined by eqn (6).6
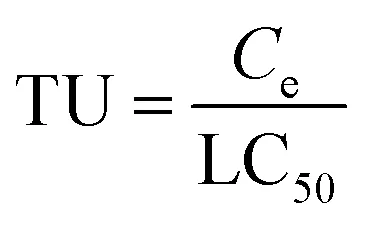


Toxic effects were further interpreted using a Hill-type concentration–response model, assuming EC_50_ = LC_50_ and a Hill coefficient (*n*) of 1, to estimate the expected mortality. All model inputs, outputs, and post-processing procedures were conducted in accordance with Organization for Economic Co-operation and Development guidelines for QSAR model development and reporting, ensuring transparency, reproducibility, and compliance with established *in silico* toxicity assessment frameworks.

## Conflicts of interest

There are no conflicts to declare.

## Supplementary Material

RA-016-D6RA05937F-s001

## Data Availability

All data supporting the findings of this study are available within the article and its supplementary information (SI) files. Detailed calculations supporting the Grunberg–Nissan mixing analysis, Taft-type reactivity relationship, and Hansen solubility parameter analysis are provided in the SI file. All other experimental results, processed datasets, and toxicity assessment outputs supporting the conclusions of this work are presented within the manuscript. Additional information is available from the corresponding author upon reasonable request. Supplementary information is available. See DOI: https://doi.org/10.1039/d6ra05937f.
