# Supplementary material for: Chemoselective depolymerization of polyurethane via solvent-engineered glycolysis
Source: RSC Adv. 2026 Aug 7;16(40):46220–33. doi: 10.1039/d6ra05937f (PMC13449149; doi:10.1039/d6ra05937f)
Supplement: Supplementary file 1 [file RA-016-D6RA05937F-s001.pdf]

Supplementary Information (SI) for RSC Advances.
This journal is © The Royal Society of Chemistry 2026

Supplementary Data

This supplementary section provides the calculations supporting the three equations used in the discussion: Equation (1) (Grunberg–Nissan mixing), Equation (2) (Taft-type reactivity relationship), and Equation (3) (Hansen solubility distance).

S1. Grunberg–Nissan analysis of the binary solvent mixtures

$$\ln(\eta_{mix}) = x_1 \ln(\eta_1) + x_2 \ln(\eta_2) + x_1 x_2 d \qquad (1)$$

System	x_{MEG}	x_{DEG}	η_{obs} (cP)	$\ln(\eta_{ideal})$	Residual	d
S1	1.0000	0.0000	45.0	3.8067	0.0000	—
S2	0.9390	0.0610	44.1	3.7931	-0.0066	-0.1151
S3	0.8724	0.1276	43.2	3.7782	-0.0123	-0.1109
S4	0.7996	0.2004	42.3	3.7619	-0.0172	-0.1071
S5	0.7195	0.2805	41.4	3.7441	-0.0208	-0.1030
S6	0.6310	0.3690	40.5	3.7243	-0.0230	-0.0989
S7	0.5327	0.4673	39.6	3.7024	-0.0236	-0.0946
S8	0.4229	0.5771	38.7	3.6779	-0.0220	-0.0903
S9	0.2994	0.7006	37.8	3.6503	-0.0180	-0.0859
S10	0.1596	0.8404	36.9	3.6191	-0.0109	-0.0814
S11	0.0000	1.0000	36.0	3.5835	0.0000	—

Note: the non-endpoint mixtures yield a small negative d value (approximately −0.096 on average), consistent with weak favorable interactions and near-ideal physical mixing.

S2. Taft-type comparison of nucleophilic reactivity

$$\log(k / k_0) = \rho\sigma + \delta E_s \qquad (2)$$

The Taft equation is written as $\log(k/k_0) = \rho\sigma^* + \delta Es$. Because only one independent pairwise rate ratio (MEG vs DEG) is available, ρ and δ cannot be uniquely determined from the present data set. The table therefore reports the observed rate ratio and the descriptor differences needed to support a qualitative steric-dominance argument.

$$log(k/k_0) = \rho\sigma^* + \delta Es$$

$$\Delta log(k/k0) = \rho\Delta\sigma^* + \delta\Delta E_s$$

Comparison	Substituent	σ*	E_s	krel	log10(krel)	Δσ*	ΔEs	Comment
MEG	–CH2CH2OH	+0.55	−0.07	1.000	0.0000	0.00	0.00	Reference nucleophile
DEG	–CH2CH2OCH2CH2OH	+0.48	−1.98	0.125	−0.9031	−0.07	−1.91	Lower intrinsic reactivity
MEG vs DEG	Pairwise contrast	—	—	0.125	−0.9031	−0.07	−1.91	Steric contrast dominates; ρ and δ are underdetermined

Calculated indices: For the MEG–DEG comparison, $\Delta\sigma^* = -0.07$ and $\Delta Es = -1.91$, giving a steric-to-electronic descriptor contrast of $|\Delta Es|/|\Delta\sigma^*| = 27.3$. The observed rate ratio corresponds to $\log10(k_{DEG}/k_{MEG}) = -0.9031$. These data support steric control qualitatively, but do not permit a unique numerical separation of ρ and δ.

Sensitivity Analysis of Sensitivity Coefficients (ρ, δ): If one assumes $\rho = 1$ only as an illustration, the implied $\delta \approx 0.473$. Conversely, if one assumes $\delta = 1$ only as an illustration, the implied $\rho \approx 12.9$. These values are not unique fit parameters and should not be reported as definitive.

S3. Hansen solubility parameter analysis

$$(R_a)^2 = 4(\delta_{d1} - \delta_{d2})^2 + (\delta_{p1} - \delta_{p2})^2 + (\delta_{h1} - \delta_{h2})^2 \qquad (3)$$

System	δ_d	δ_p	δ_h	R_a	Glycolysate η (cP)	Δη vs S1	Comment
S1	17.0	11.0	26.0	17.58	50	0	Reference
S2	16.92	11.37	25.45	17.22	38	-12	Lower viscosity
S3	16.84	11.74	24.9	16.87	31	-19	Minimum observed viscosity
S4	16.76	12.11	24.35	16.55	35	-15	
S5	16.68	12.48	23.8	16.25	42	-8	Selected optimum
S6	16.6	12.85	23.25	15.98	52	+2	
S7	16.52	13.22	22.7	15.73	67	+17	
S8	16.44	13.59	22.15	15.5	75	+25	
S9	16.36	13.96	21.6	15.31	95	+45	
S10	16.28	14.33	21.05	15.14	110	+60	
S11	16.2	14.7	20.5	15.0	102	+52	

Supplementary Figures

Fig. S1

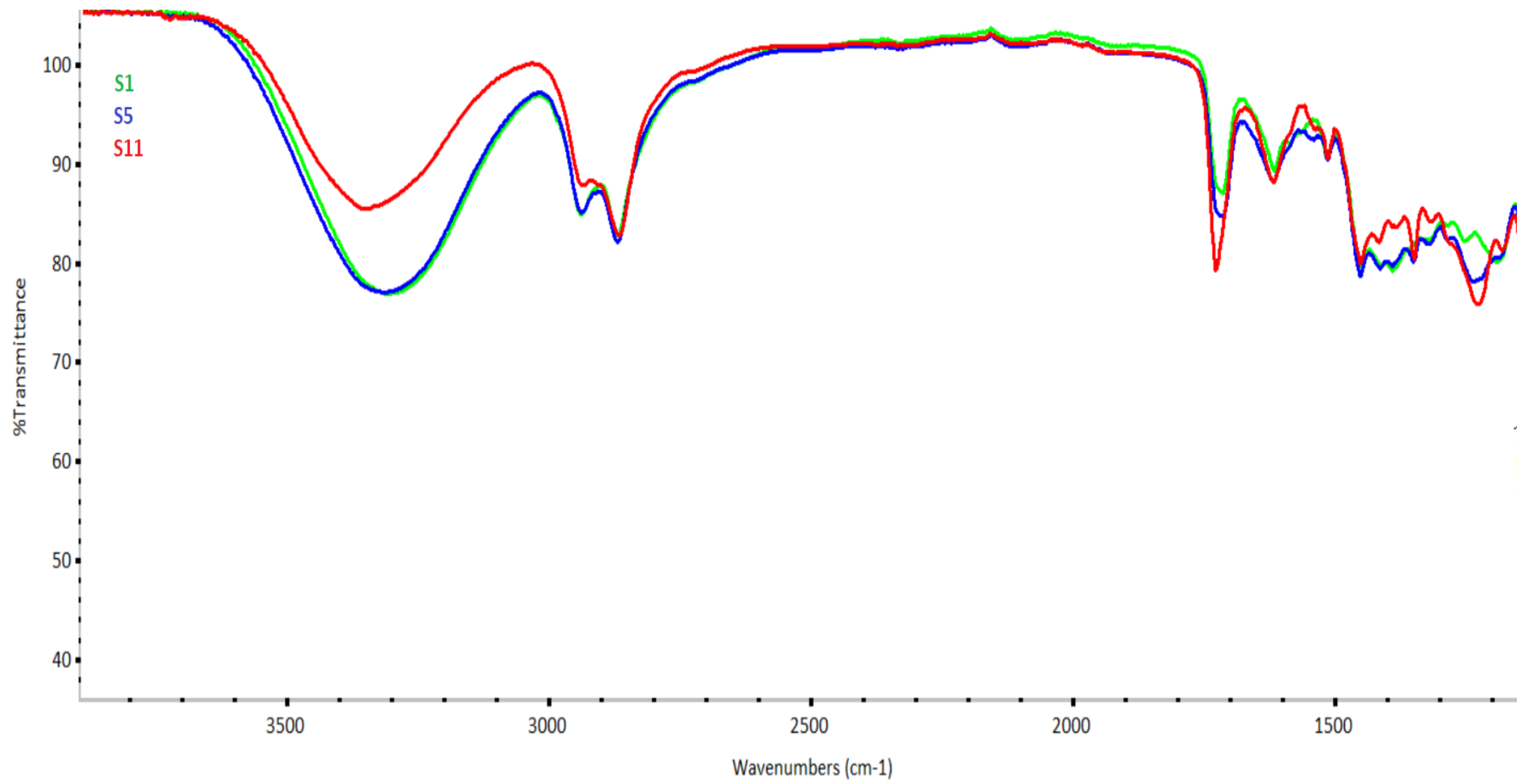

Figure S1: Comparative analysis of MEG-S1, DEG--S11, and optimal binary mixture of MEG-DEG using FTIR

Fig. S2

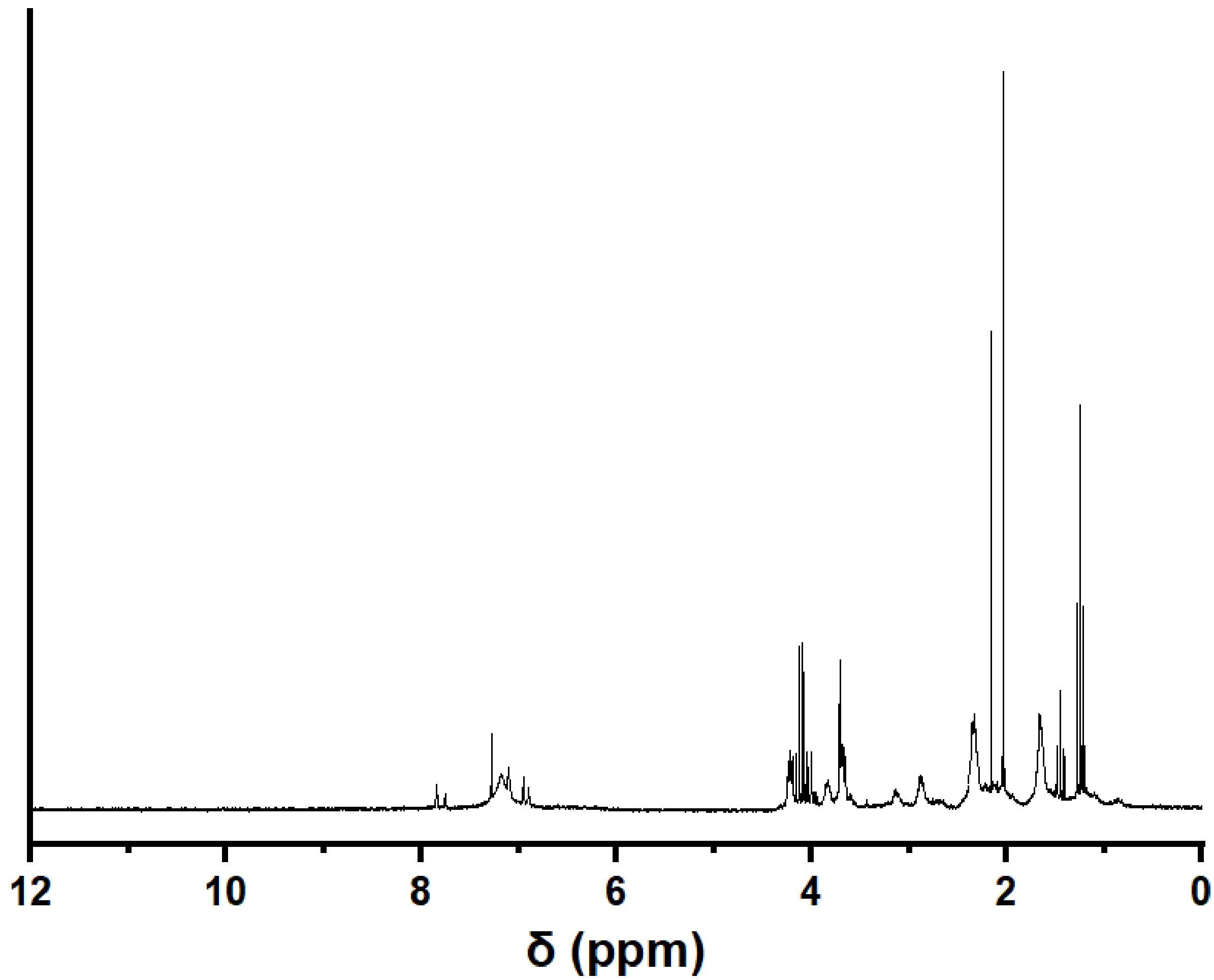

Figure S2: ^{1}H NMR spectrum of the purified glycolysate obtained under the optimized glycolysis conditions.
